# Targeting STAT3 prevents bile reflux‐induced oncogenic molecular events linked to hypopharyngeal carcinogenesis

**DOI:** 10.1111/jcmm.17011

**Published:** 2021-12-01

**Authors:** Dimitra P. Vageli, Panagiotis G. Doukas, Athanasios Siametis, Benjamin L. Judson

**Affiliations:** ^1^ The Yale Larynx Laboratory Department of Surgery (Otolaryngology) Yale School of Medicine New Haven Connecticut USA

**Keywords:** bile reflux, gastro‐oesophageal reflux, head and neck cancer, hypopharyngeal cancer, laryngopharyngeal reflux, Nifuroxazide, SI3‐201, STA‐21, STAT3, STAT3 inhibition

## Abstract

The signal transducer and activator of transcription 3 (*STAT3*) oncogene is a transcription factor with a central role in head and neck cancer. Hypopharyngeal cells (HCs) exposed to acidic bile present aberrant activation of STAT3, possibly contributing to its oncogenic effect. We hypothesized that STAT3 contributes substantially to the bile reflux‐induced molecular oncogenic profile, which can be suppressed by *STAT3* silencing or pharmacological inhibition. To explore our hypothesis, we targeted the STAT3 pathway, by knocking down *STAT3* (STAT3 siRNA), and inhibiting STAT3 phosphorylation (Nifuroxazide) or dimerization (SI3‐201; STA‐21), in acidic bile (pH 4.0)‐exposed human HCs. Immunofluorescence, luciferase assay, Western blot, enzyme‐linked immunosorbent assay and qPCR analyses revealed that *STAT3* knockdown or pharmacologic inhibition significantly suppressed acidic bile‐induced STAT3 activation and its transcriptional activity, Bcl‐2 overexpression, transcriptional activation of *IL6*, *TNF*‐*α*, *BCL2*, *EGFR*, *STAT3*, *RELA(p65)*, *REL* and *WNT5A*, and cell survival. Our novel findings document the important role of STAT3 in bile reflux‐related molecular oncogenic events, which can be dramatically prevented by *STAT3* silencing. STA‐21, SI3‐201 or Nifuroxazide effectively inhibited STAT3 and cancer‐related inflammatory phenotype, encouraging their single or combined application in preventive or therapeutic strategies of bile reflux‐related hypopharyngeal carcinogenesis.

## 1 INTRODUCTION

The carcinogenic effect of bile reflux in hypopharyngeal mucosa has been recently documented by our established *in vitro* and *in vivo* models.^1^, ^2^, ^3^, ^4^, ^5^, ^6^, ^7^ We have shown that acidic bile (BA) can cause a progressive mutagenic effect characterized by a dramatic activation of a characteristic molecular phenotype,^2^, ^3^, ^4^ assigned as ‘BA‐induced mRNA oncogenic phenotype’, including central molecules in head and neck squamous cell carcinoma (HNSCC), such as nuclear factor kappa B (NF‐κB) and signal transducer and activator of transcription 3 (STAT3).^8^, ^9^, ^10^, ^11^ Exploration of the BA effect using pharmacologic and dietary inhibitors of NF‐κB revealed the key role of NF‐kB in this process by activating early neoplastic molecular events.^12^, ^13^, ^14^, ^15^, ^16^, ^17^, ^18^ However, the role of STAT3 in bile reflux‐related hypopharyngeal carcinogenesis remains unclear. We hypothesized that STAT3 contributes substantially to the BA‐related oncogenic effect, by inducing transcriptional activation of inflammatory and cancer‐related genes, according to previously established BA‐induced ‘mRNA oncogenic phenotype’,^1^, ^2^, ^3^, ^4^, ^5^, ^6^, ^7^, ^8^, ^12^, ^13^, ^14^, ^15^, ^16^, ^17^, ^18^ in exposed human hypopharyngeal primary cells (HCs) and preserving cell survival.

The STAT3 is considered an oncogene^8^, ^19^ and its upregulation has been associated with development and progression of head and neck cancer,^9^, ^20^, ^21^ and especially of HPV‐negative HNSCC.^10^ The mechanism by which STAT3 is activated has been described by others.^8^, ^22^, ^23^ STAT3 proteins, as transducers of cytoplasmic signals from extracellular stimuli, including cancer‐related cytokines and growth factors, can be activated through Janus‐kinase family members (JAK).^24^, ^25^ JAKs after their dimerization can phosphorylate a tyrosine residue (Tyr705) of STAT3 within its Src homology 2 (SH2) domains. Then, STAT3 can homodimerize or heterodimerize with other STAT proteins to translocate to the nucleus and activate the transcription of inflammatory and cancer‐related genes.^26^, ^27^ JAK/STAT3 activation plays a crucial role in inflammatory‐related carcinogenesis.^8^, ^25^, ^26^ EGFR can also activate STAT3 by its intrinsic tyrosine kinase activity.^9^, ^27^ IL‐6/STAT3 signalling^11^ has also been previously suggested to play a role in bile reflux‐related oncogenesis, through NF‐κB activated pathway.^12^, ^13^, ^14^, ^15^, ^16^, ^17^, ^18^

To investigate whether STAT3 actually contributes to early oncogenic molecular events, previously linked to bile‐induced hypopharyngeal carcinogenesis,^2^, ^4^ we targeted the STAT3 pathway by knocking down *STAT3* gene expression, as well as by inhibiting its activation using specific STAT3 pharmacologic inhibitors. STAT3 pharmacologic inhibition consisted of blocking (i) the upstream extracellular receptor JAK2, which affects STAT3 phosphorylation; or (ii) the SH2 domain of phosphorylated STAT3, which blocks its dimerization and DNA binding, and therefore preventing the subsequent target‐gene transcription.^22^, ^25^, ^28^, ^29^ Identification of the mechanism by which STAT3 affects acidic bile‐induced oncogenic molecular events may not only contribute to the characterization of its role in hypopharyngeal carcinogenesis but may also be more important to the development of new effective targeted agents in the prevention and therapy of this process.

## 2 MATERIALS AND METHODS

### 2.1 Cell culture and treatment conditions

Normal human hypopharyngeal cells (HCs; Celprogen Inc.) were cultured, in Human Hypopharyngeal Normal Cell Culture Media (Celprogen Inc.), at 37°C in humidified air and 5% CO_2_, as previously described.^7^, ^13^, ^16^ An intermittent exposure of HCs was performed, to experimental and control media for 7 min, twice per day, for 4–5 days, as previously described.^9^ Experimental media included (i) ‘BA’, acidic bile at pH 4.0, (ii) ‘Nif’, BA plus 10 μM Nifuroxazide (CAS 965‐52‐6; Santa Cruz Biotechnology Inc.), (iii) ‘SI3‐201’, BA plus 50 μM STAT3 Inhibitor VI, S3I‐201 (CAS 19983‐44‐9; Santa Cruz Biotechnology Inc.) and (iv) ‘STA‐21’, BA plus 20 μM STA‐21 (CAS 28882‐53‐3; Santa Cruz Biotechnology Inc.; Supplementary Material; Table S1). All experimental groups were repetitively exposed to 400 μM of a mixture of conjugated primary bile acids (Sigma Aldrich) as previously described,^1^, ^7^, ^13^, ^16^ in serum‐free medium (Dulbecco modified Eagle's medium/F12, 1% pen/strep, Gibco^®^). pH at 4.0 was adjusted by 1 M HCl (using a pH meter). ‘BA’ or acidic bile at pH 4.0, the cut‐off of reflux disease^30^ was used according to our prior findings.^1^, ^2^, ^3^, ^4^, ^7^ Control media included serum‐free medium, as used in experimental groups, at either pH 4.0 (acid control) or neutral pH 7.0 (control including vehicle) (Supplementary material; Table S1). After each treatment, experimental or control media were replaced by serum‐free media until the next exposure cycle.

Experimental and control groups were cultured in parallel. All the experiments were independently repeated three times. Cells were harvested immediately after the last treatment by 0.05% trypsin‐EDTA (Gibco^®^).

### 2.2 STAT3 knockdown

To knockdown *STAT3*, STAT3 siRNA (STAT3 siRNA(h); sc‐29493, Santa Cruz Biotechnology Inc.) and Control siRNA, as a negative control (Control siRNA‐A; sc‐37007, Santa Cruz Biotechnology Inc.) were used. STAT3 and Control siRNAs were diluted to a final concentration of 5 nM in serum‐free culture medium (Opti‐MEM^®^ serum‐reduced growth medium, Gibco™ by Thermo Fisher Scientific), including HiPerFect^®^Transfection Reagent (3 μl/well; Qiagen), according to the manufacturers’ instructions, and incubated for 10 min at room temperature. Cells were mixed with transfected complexes, seeded at 1.5 × 10^5^ cells/well of six‐well plates and incubated under normal growth conditions (at 37°C and 5% CO_2_) (Supplementary Material; Table S2).

Sixteen hours after transfection, media were changed with complete growth medium and 4 h later were treated with ‘BA’ for 7 min. The media were removed and replaced with serum‐free medium until the next day, we repeated the 7‐min treatment two times with an interval time 6 h. After 12 h, cells were treated for 7‐min and immediately after this last treatment media were removed, the cells were washed once with PBS and harvested either (i) for total protein isolation, using M‐PER reagent (mammalian protein extraction reagent; Thermo Scientific), or (iii) for total RNA isolation using RNA mini kit (Qiagen). Assays were carried out according to the manufacturer's instructions and performed in triplicate. All experiments were independently repeated two times.

### 2.3 Luciferase assay

Luciferase assay was performed to measure the transcriptional activity of STAT3 dimers (homodimers or heterodimers) in HCs exposed to (a) BA with knockdown of STAT3 or (b) BA with pharmacologic inhibitors of STAT3, Nifuroxazide, S3I‐201 or STA‐21, compared to BA alone and controls, as described in Supplementary Material (Tables S1 and S2). STAT3 dual‐luciferase reporter assay was used (Cignal reporter assay by Qiagen), including (i) a firefly luciferase reporter for STAT3 and a constitutively expressing Renilla luciferase construct (Creport‐STAT3), and (ii) a cignal negative control with a non‐inducible reporter construct and a constitutively expressing Renilla luciferase construct (Creport‐NC). A reverse transfection was performed, using Lipofectamine^®^ 2000 (Invitrogen™), according to manufacturer's procedure.

### 2.4 Immunofluorescence cell staining

Immunofluorescence (IF) assay was performed for p‐STAT3 (Tyr705) and p‐NF‐κB (p65 S536), as previously described^9^, ^10^ and in Supplementary Material. Briefly, HCs were grown on multiwall chamber slides (Lab‐Tek^®^; Thermo Fisher Scientific) and treated with BA, with or without pharmacologic inhibitors of STAT3, Nifuroxazide, SI3‐201 or STA‐21, acid at pH 4.0 alone. Primary antibodies were used, including anti‐p‐STAT3 (Tyr705) (rabbit mAb, D3A7 XP^®^, Cell Signaling Technology, Inc.) or anti‐phospho‐NF‐*κ*B (rabbit polyclonal anti‐phospho‐p65 Ser536, AbD Serotec, BIO‐RAD). Secondary antibodies were also used, including anti‐rabbit or anti‐mouse DyLight^®^488 (green; Vector Labs). Prolong Gold Mountant with diamidino‐phenylindole (ProLong^®^ Diamond Antifade Mountant with DAPI; Life Technologies, Thermo Scientific) was also used for nuclear staining (blue colour).

Zeiss Confocal microscope and imaging software by Zen were used to examine stained slides and captured images, respectively (Zen imaging software, Carl Zeiss, microscopy GmbH).^9^, ^10^ Expression levels of p‐STAT3 and p‐NF‐κB were assessed by fluorescence intensity (mean ± SD bin count) from at least two intendent images (>10 cells; Zen imaging software).

### 2.5 Protein expression analysis

Western blot analysis and a direct enzyme‐linked immunosorbent assay (ELISA) were performed, as previously described^7^, ^13^, ^16^, ^31^ and in Supplementary Material, to monitor the successful knockdown of *STAT3* expression, and to determine the effect of *STAT3* knockdown or its pharmacologic inhibition on BA‐induced STAT3, NF‐*κ*B and Bcl‐2 protein levels. We used primary antibodies for p‐STAT3 (Tyr 705) (clone B‐7), STAT3 (clone F‐2), p‐NF‐*κ*B (p65 Antibody 27. Ser 536), bcl2 (Clone N‐19), Histone 1 (AE‐4) and β‐actin (C4) (Santa Cruz Biotechnology Inc.). Protein levels obtained by Western blot analysis were quantified by the Gel imaging system (Bio‐Rad) in each nuclear or cytoplasmic cellular compartment (Image Lab 5.2 analysis software, Bio‐Rad). Protein levels obtained by ELISA were quantified by Gen5™ software reading the absorbance values using a microplate reader (Sunergy1, BIOTEK; Gen5™ software; BioTek Instruments Inc.). Assays were carried out according to the manufacturer's instructions and performed in triplicates and repeated two times, independently.

### 2.6 Quantitative real‐time polymerase chain reaction

Quantitative real‐time polymerase chain reaction (qPCR) analysis (Bio‐Rad real‐time thermal cycler CFX96™; Bio‐Rad) was performed, as previously described^7^, ^13^, ^16^ and in Supplementary Material, to evaluate the effect of STAT3 knockdown or pharmacologic inhibition on transcriptional levels of *EGFR*, *TNF*‐*α*, *IL6*, *STAT3*, *RELA(p65)*, *REL*, *BCL2* and *WNT5A*, previously associated with HNSCC^32^, ^33^, ^34^, ^35^, ^36^, ^37^, ^38^, ^39^, ^40^ and in particular with bile carcinogenesis^2^, ^3^, ^4^, ^5^ and HSCC.^41^ Specific primers were used for target genes and reference housekeeping gene, human glyceraldehyde 3‐phosphate dehydrogenase (*hGAPDH*) (QuantiTect Primers Assays; Qiagen; Supplementary Materials; Table S3) and data were analysed by CFX96™ software.^7^, ^13^, ^16^ Relative mRNA expression levels were estimated for each target gene compared to the reference control gene (ΔΔ*C*t).

### 2.7 Cell viability assay

Cell Titer‐Glo^®^ Luminescent Cell Viability Assay (Promega Corp.) was used as described in Supplementary Material. All values were normalized to mean value of untreated controls. All experimental groups and controls were performed in triplicates.

### 2.8 Statistical analysis

GraphPad Prism 7 software and multiple *t* test analysis (GraphPad Prism 7 software; *t* test; multiple comparisons by Holm‐Sidak; Graph Pad Prism 7.0, GraphPad Software Inc.) were used to demonstrate the differential expression (*p*‐values < 0.05) for each analysed gene, protein expression and cell viability between different experimental and control groups.

## 3 RESULTS

### 3.1 Knockdown of STAT3 reduces BA‐induced total STAT3, p‐STAT3 and Bcl‐2 protein levels

**FIGURE 1:**
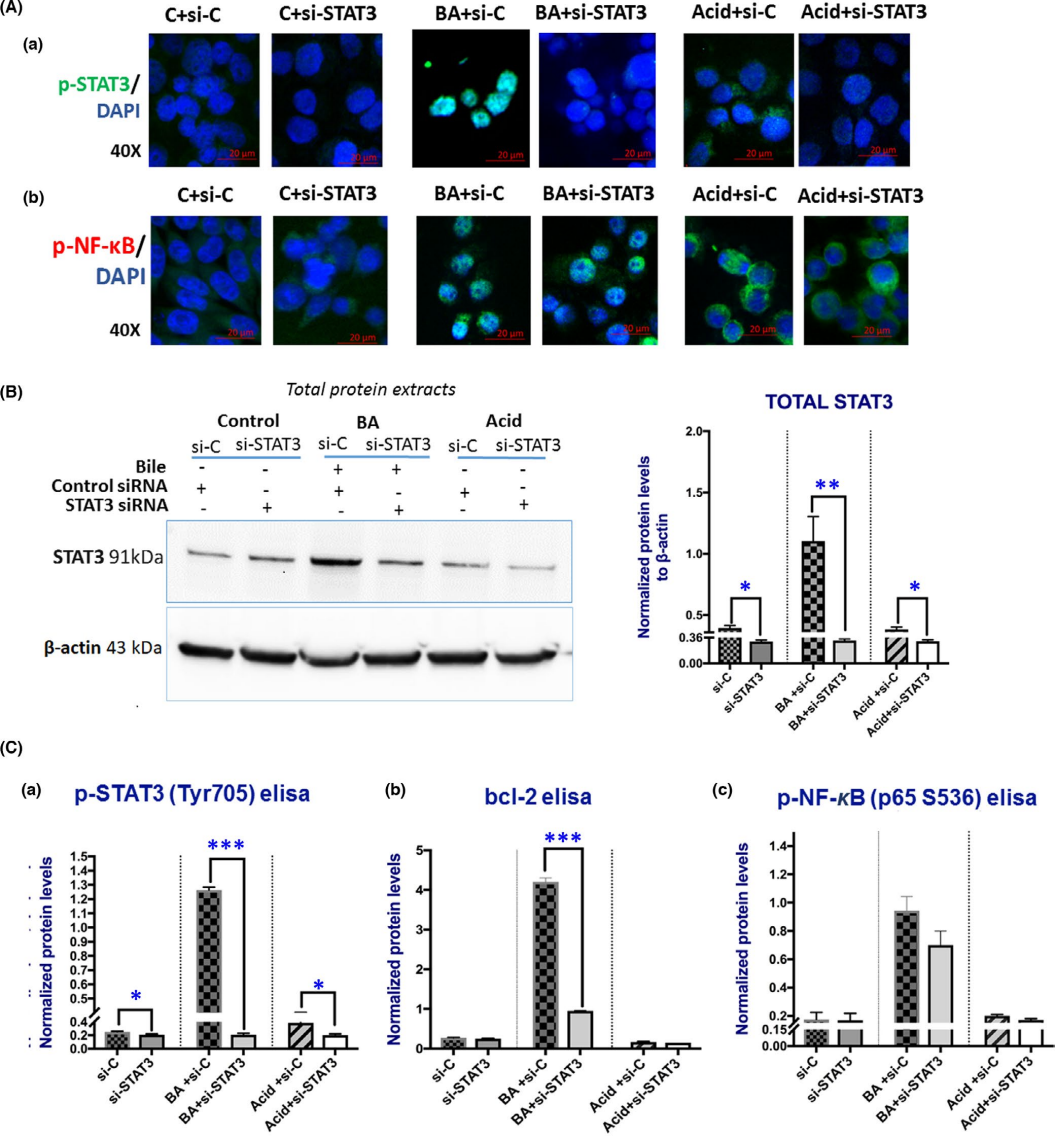
Silencing of *STAT3* suppressed total STAT3, p‐STAT3 (Tyr705) and bcl2 protein levels, with a minimal suppressive effect on p‐NF‐κB (p65 S536), in BA‐treated HCs. (A) (a) Immunofluorescence staining for p‐STAT3 (Tyr705) (green: p‐STAT3; blue: nuclear DNA staining with DAPI; scale bar 20 μm; Zen imagining software). (b) Immunofluorescence staining for p‐NF‐κB (p65 S536) (green: p‐ NF‐κB (p65 S536); blue: nuclear DNA staining with DAPI; scale bar 20 μm; Zen imagining software). (B) Western blot analysis for total STAT3 in BA‐treated HCs and controls (Control: media at pH 7.0, including vehicle; Acid: media at pH 4.0) with knockdown of *STAT3* gene. Graph depicts the total STAT3 protein levels, in BA and control‐treated HCs after STAT3 knockdown. (C) Graphs depict the total protein levels, by ELISA, of p‐STAT3 (Tyr705), Bcl‐2 and p‐NF‐κB (p65 S536) in BA‐treated HCs after STAT3 knockdown. (from left to right) *si*‐*C*: media at pH 7.0 plus Control siRNA; *si*‐*STAT3*: media at pH 7.0 plus STAT3 siRNA; *BA*+*si*‐*C*: Bile at pH 4.0 plus Control siRNA; *BA*+*si*‐*STAT3*: Bile at pH 4.0 plus STAT3 siRNA; *Acid*+*si*‐*C*: media at pH 4.0 plus Control siRNA; *Acid*+*si*‐*STAT3*: media at pH 4.0 plus STAT3 siRNA (β‐actin was used to normalize total protein extracts; *t* test; multiple comparisons by Holm‐Sidak, **p < 0.005; ***p < 0.0005; GraphPad Prism 7.0; means ± SD of three independent experiment)

Immunofluorescence (IF) assay revealed that *STAT3* knockdown prevented the BA‐induced activation of STAT3, as indicated by weak nuclear straining of p‐STAT3 (Tyr705) in treated HCs compared to BA alone (Figure 1A‐a). IF also revealed that *STAT3* knockdown had a minimal effect on BA‐induced activation of NF‐κB, as shown by a less intense nuclear staining of p‐NF‐κB (p65 S536) relative to BA alone (Figure 1A‐b). Western blot analysis confirmed that silencing of *STAT3* effectively suppressed STAT3 protein levels produced by BA exposure in HCs (Figure 1B). Quantification by ELISA also showed that *STAT3* knockdown significantly reduced the BA‐induced total p‐STAT3 (Tyr705; Figure 1C‐a) and Bcl‐2 protein levels (Figure 1C‐b). *STAT3* knockdown also induced a decrease, although not statistically significant, in total p‐NF‐κB levels, compared to BA alone (Figure 1C‐c). IF, Western blot and ELISA analyses showed that controls produced low levels of STAT3 and its activated form (p‐STAT3), and so silencing of *STAT3* induced slight changes (Figure 1A‐a,B,C‐a). Similarly, controls presented low p‐NF‐κB (p65 S536) levels, mostly located in the cytoplasm (Figure 1A), accompanied by low Bcl‐2 levels, while silencing of *STAT3* had a minimal effect on their expression (Figure 1B,C‐b,c).

### 3.2 Pharmacologic inhibition of STAT3 prevents BA‐induced STAT3 activation and Bcl‐2 overexpression

**FIGURE 2:**
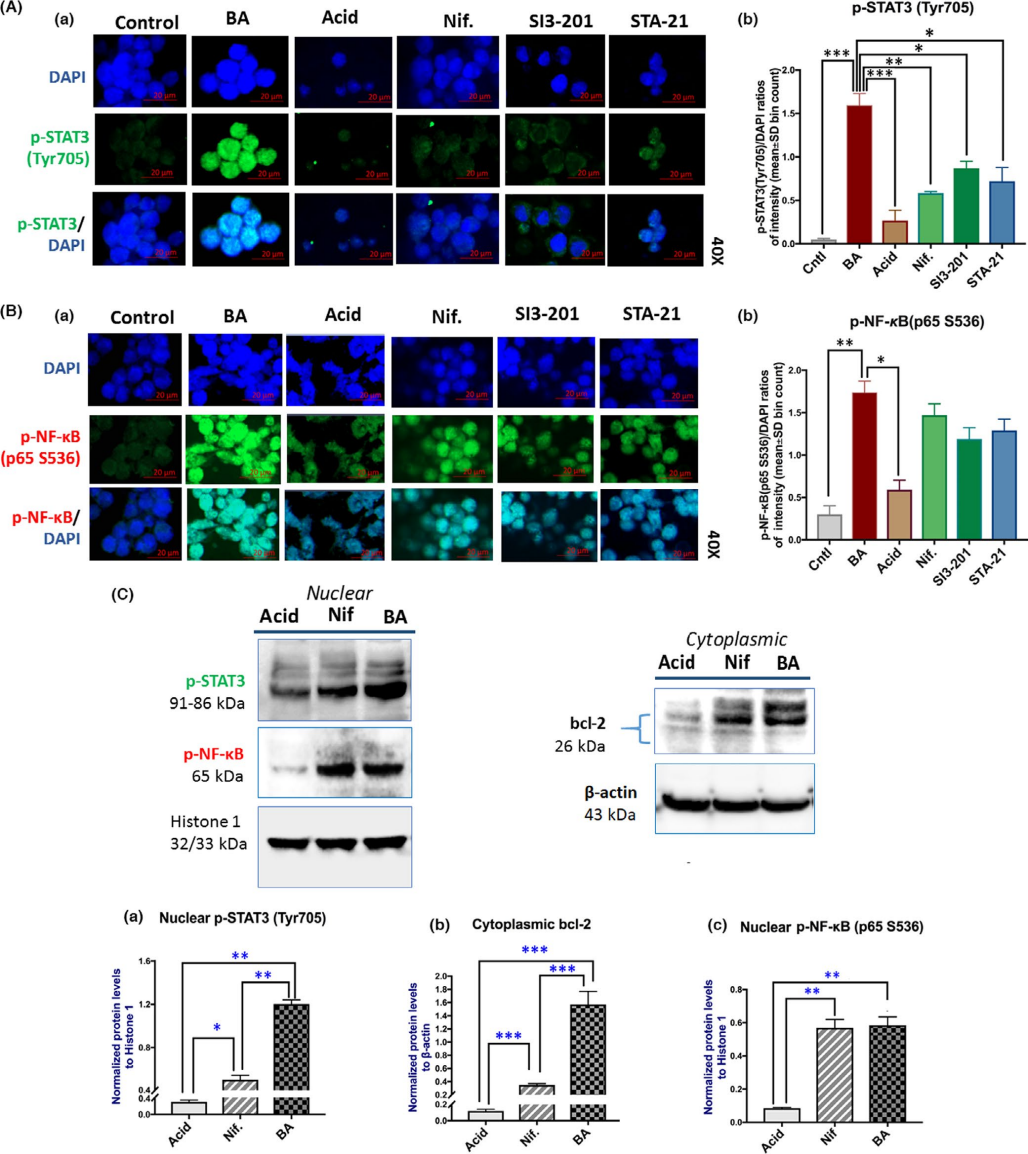
Pharmacologic inhibition of STAT3 prevents BA‐induced nuclear localization of p‐STAT3 and Bcl‐2 overexpression, with a minimal effect on p‐NF‐κB activation, in treated HCs. (A) (a) Immunofluorescence staining for p‐STAT3 (Tyr705) (green: p‐STAT3; blue: nuclear DNA staining with DAPI; scale bar 20 μm; Zen imagining software). (b) Graph depicts the nuclear protein levels of p‐STAT3 (Tyr705) in treated HCs. (B) (a) Immunofluorescence staining for p‐NF‐κB (p65 S536) (green: p‐ NF‐κB (p65 S536); blue: nuclear DNA staining with DAPI; scale bar 20 μm; Zen imagining software). (b) Graphs created depict the nuclear protein levels of p‐NF‐κB (p65 S536) in treated HCs. (from left to the right). *Control*: media at pH 7.0 (including vehicle); *BA*: acidic bile (pH 4.0); *Acid*: media at pH 4.0; *Nif*: BA plus Nifuroxazide; *SI3*‐*201*: BA plus STAT3 inhibitor VI (S3I‐201); *STA*‐*21*: BA plus STA‐21. (C) Graphs depict the (a) nuclear protein levels of p‐STAT3 (Tyr705), (b) cytoplasmic levels of Bcl‐2 and (c) nuclear levels of p‐NF‐κB (p65 S536), in BA‐treated HCs, with or without pharmacologic inhibition of STAT3, by Western blot analysis. *Acid*: media at pH 4.0; *Nif*: acidic bile plus Nifuroxazide. *BA*: acidic bile (H 4.0); (Histone 1 and β‐actin were used to normalize nuclear and cytoplasmic protein extracts, respectively; by Image Lab 5.2 analysis software, Bio‐Rad; *t* test; multiple comparisons by Holm‐Sidak, *p < 0.05; **p < 0.005; ***p < 0.0005; GraphPad Prism 7.0; means ± SD of three independent experiment)

Immunofluorescence (IF) assay presented that BA induced an intense nuclear staining of p‐STAT3 (Tyr705), as it was expected.^7^, ^14^ However, application of Nifuroxazide, which blocks the upstream extracellular receptor JAK2, affecting STAT3 phosphorylation,^28^ or application of SI3‐201 or STA‐21 that block the SH2 domain of phosphorylated STAT3, inhibiting dimerization and DNA binding,^22^, ^29^ successfully suppressed BA‐induced activation of STAT3, as shown by a less intense nuclear staining of p‐STAT3 (Tyr 705) in treated HCs, compared to BA alone (Figure 2A‐a). Scoring of IF staining revealed significantly higher nuclear levels of p‐STAT3 (Tyr705) in BA‐treated HCs, compared to controls (Figure 2A‐b). The application of Nifuroxazide, SI3‐201 or STA‐21 resulted in significantly lower nuclear levels of p‐STAT3, compared to BA. In particular, Nifuroxazide was found to induce the lowest p‐STAT3 nuclear levels among SI3‐201 and STA‐21 (Figure 2A‐b).

IF assay also revealed that application of Nifuroxazide, SI3‐201 or STA‐21, diminished the BA‐induced activation of NF‐κB, as shown by a less intense nuclear staining of p‐NF‐κB (p65 S536) relative to BA alone (Figure 2B‐a). However, scoring of IF staining did not reveal a significant difference between p‐NF‐κB (p65 S536) nuclear levels measured in HCs exposed to BA plus STAT3 pharmacologic inhibitors, compared to BA alone (Figure 2B‐b).

In order to confirm the above IF data, and further determine if targeting JAK/STAT3 phosphorylation can affect the acidic bile‐induced STAT3 activation, Bcl‐2 overexpression and NF‐κB activation, we performed Western blot analysis. Our analysis revealed that Nifuroxazide inhibited the activation of STAT3 and Bcl‐2 overexpression, caused by acidic bile. This was shown by significantly reduced nuclear levels of p‐STAT3(Tyr705) and cytoplasmic levels of Bcl‐2 in HCs exposed to BA plus Nifuroxazide compared to BA alone (Figure 2C‐a,b). However, our analysis showed that Nifuroxazide did not reduce BA‐induced nuclear levels of p‐NF‐κB (Figure 2C‐c).

### 3.3 STAT3 knockdown or pharmacologic inhibition of STAT3 reduces BA‐induced STAT3 transcriptional activity

**FIGURE 3:**
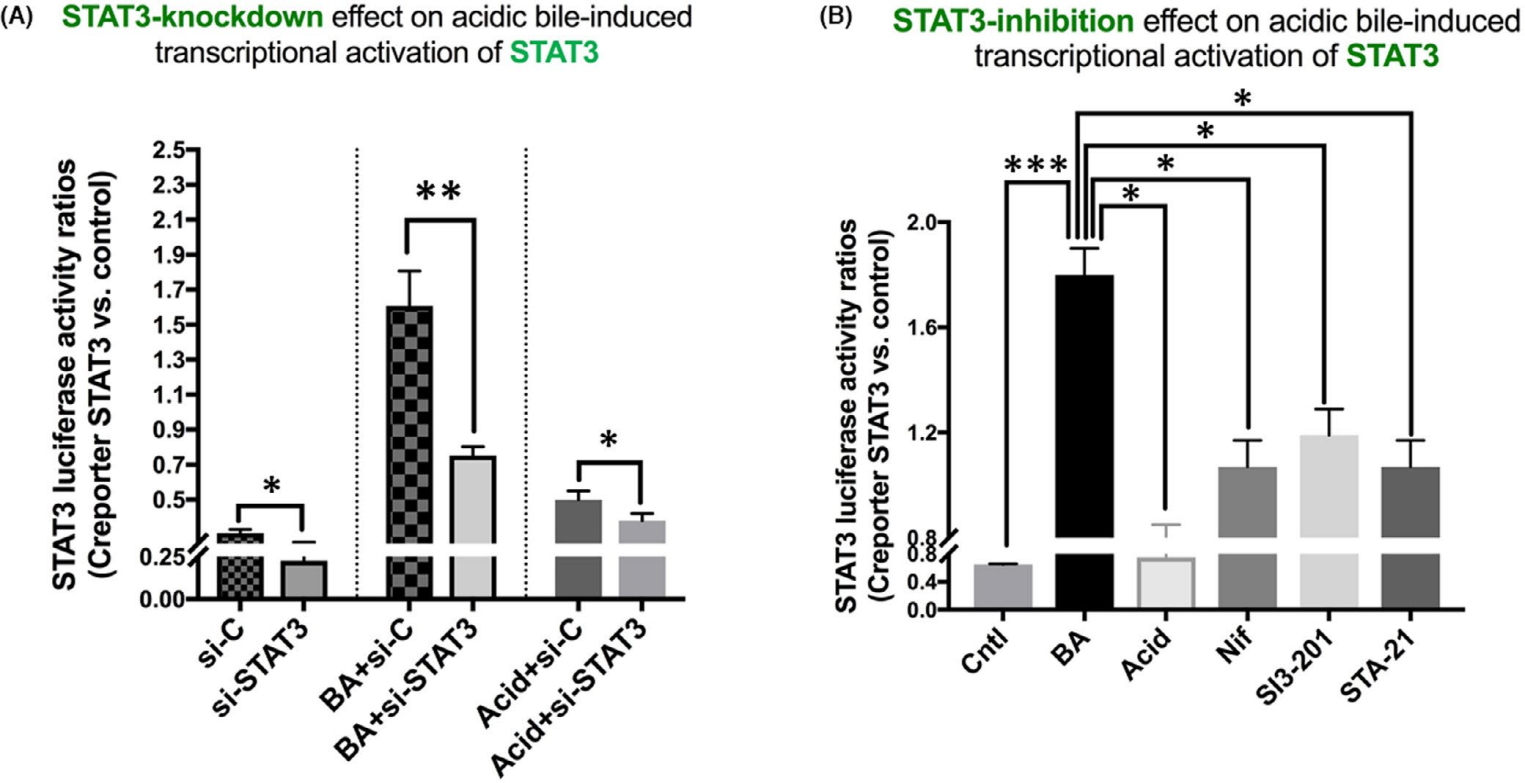
Luciferase assay for STAT3 transcriptional activity in BA‐treated HCs, with targeting STAT3 pathway. Columns represent ratios of STAT3 luciferase transcriptional activity in HCs transfected with STAT3 luciferase responsive element (Creport‐STAT3) versus luciferase activity in HCs transfected with control luciferase reporter (Creport‐control) (A) *STAT3 knockdown*. (from left to right) *si*‐*C*: media at pH 7.0 plus Control siRNA; *si*‐*STAT3*: media at pH 7.0 plus STAT3 siRNA; *BA*: Bile at pH 4.0 plus Control siRNA; *BA*+*si*‐*STAT3*: Bile at pH 4.0 plus STAT3 siRNA; *Acid*: media at pH 4.0 plus Control siRNA; *Acid*+*si*‐*STAT3*: media at pH 4.0 plus STAT3 siRNA. (B) *Pharmacologic inhibition of STAT3*. (from left to right) *Cntl*: media at pH 7.0 (including vehicle); *BA*: acidic bile (pH 4.0); *Acid*: media at pH 4.0; *Nif*: BA plus Nifuroxazide; *SI3*‐*201*: BA plus STAT3 inhibitor VI (SI3‐201); *STA*‐*21*: acidic bile plus STA‐21. (*t* test; multiple comparisons by Holm‐Sidak, **p < 0.005; ***p < 0.0005; GraphPad Prism 7.0; means ± SD of three independent experiment)

Our analysis revealed a significant reduction of STAT3‐reporter luciferase activity in BA‐treated HCs with *STAT3* knockdown, compared to BA alone (BA plus STAT3 siRNA vs. BA plus Control siRNA; Figure 3A). Acid and neutral control‐treated groups appeared with low levels of STAT3 luciferase activity; however, silencing of *STAT3* gene also suppressed its levels (STAT3 siRNA vs. Control siRNA).

Our analysis, using STAT3‐reporter luciferase assay and Nifuroxazide, SI3‐201 or STA‐21, also revealed that pharmacologic inhibition of STAT3 affected the BA‐induced STAT3 transcriptional activity in HCs, as similarly shown by knocking down of *STAT3*. Specifically, our analysis showed that BA‐treated HCs presented significantly higher STAT3‐transcriptional activity, compared to controls (Figure 3B). However, application of all three pharmacologic inhibitors significantly reduced BA‐induced STAT3‐transcriptional activity. This was indicated by significantly lower levels of luciferase activity for STAT3‐reporter, in HCs exposed to BA plus STAT3 pharmacologic inhibitors, compared to BA alone (Figure 3B).

### 3.4 STAT3 knockdown suppresses transcriptional changes caused by acidic bile

**FIGURE 4:**
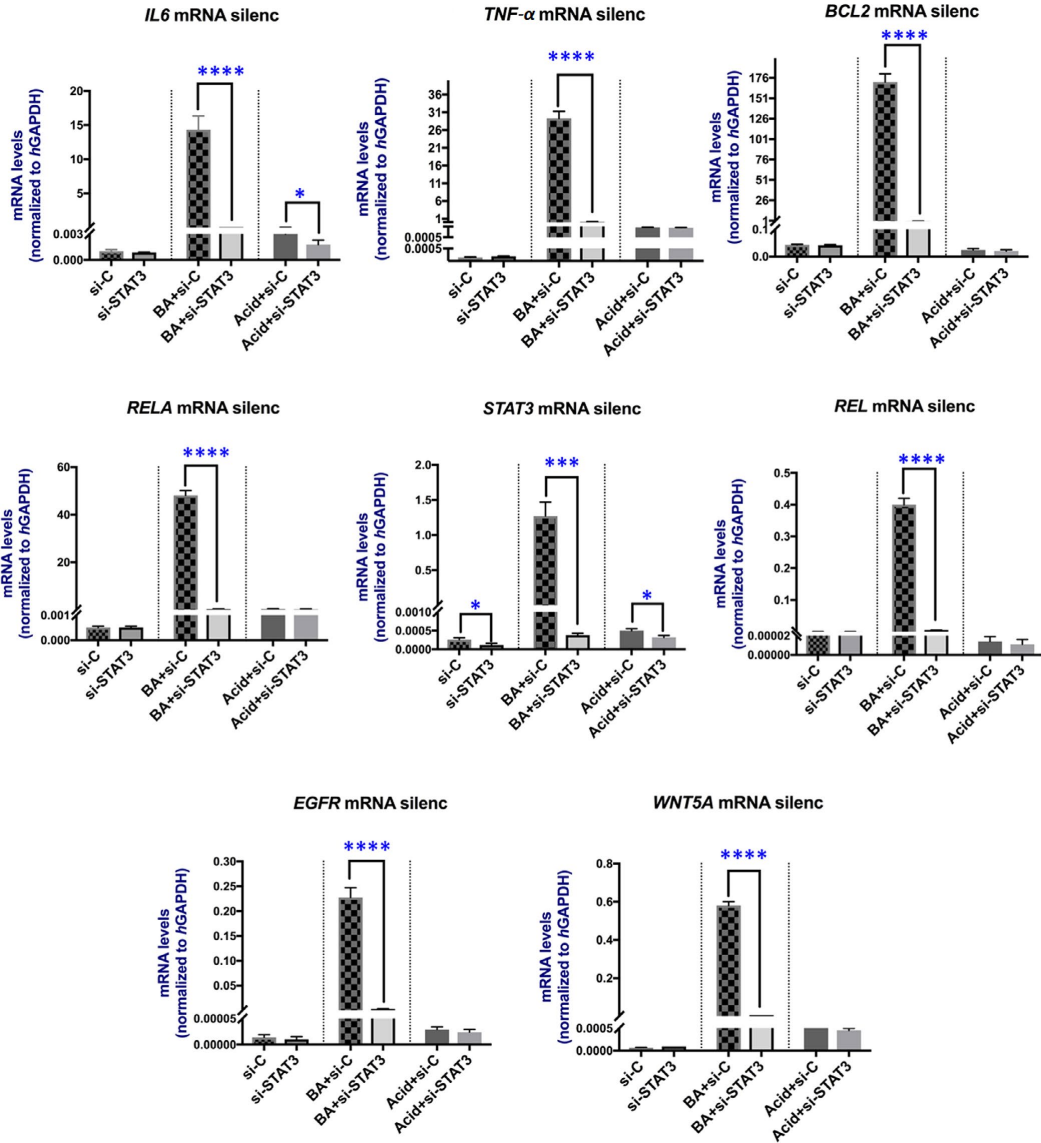
Silencing of *STAT3* suppressed BA‐induced transcriptional changes. Columns represent mRNA levels of each analysed gene in treated groups with knockdown of *STAT3* (mRNA silenc) by qPCR (normalized mRNAs to *hGAPDH* reference control) (from left to right) *si*‐*C*: media at pH 7.0 plus Control siRNA; media at pH 7.0 plus *si*‐*STAT3*: STAT3 siRNA; *BA*: Bile at pH 4.0 plus Control siRNA; *BA*+*si*‐*STAT3*: Bile at pH 4.0 plus STAT3 siRNA; *Acid*: media at pH 4.0 plus Control siRNA; Acid+si‐STAT3: media at pH 4.0 plus STAT3 siRNA. (*t* test; multiple comparisons by Holm‐Sidak, *p < 0.05; **p < 0.005; ***p < 0.0005; ****p < 0.00005; GraphPad Prism 7.0; means ± SD of three independent experiment)

qPCR analyses showed that knockdown of *STAT3* reversed the BA‐induced transcriptional changes of genes, previously assigned as BA‐induced ‘mRNA oncogenic phenotype’ and linked to malignant transformation of murine hypopharyngeal mucosa caused by BA and HNSCC^2^, ^4^, ^7^, ^8^, ^9^, ^11^, ^12^, ^13^, ^26^, ^27^, ^32^, ^33^, ^34^, ^35^, ^36^, ^37^, ^38^, ^39^, ^40^, ^41^ (Figure 4). As shown in Figure 4 and Table 1, BA‐treated HCs with *STAT3* knockdown produced significantly lower mRNA levels of *IL6*, *TNF*‐*α*, *BCL2*, *RELA(P65)*, *STAT3*, *REL*, *WNT5A* and *EGFR*, compared to BA alone (Supplementary Material; Table S4). Acid and neutral control groups presented with low mRNA levels of the analysed genes and *STAT3* knockdown had a minimal effect on them, except *STAT3* and *IL6* in acid control group (Supplementary Material; Table S4).

**TABLE 1:** The effect of STAT3‐inhibition on oncogenic mRNA phenotype caused by acidic bile in HCs

	Nifuroxazide^a^	SI3‐201^a^	STA‐21^a^	siSTAT3^a^
*IL6*	−102	−600	−1554	−313
*TNF‐α*	−5.1	−3.4	−4	−234
*BCL2*	−9	−3.5	−2.8	−285
*STAT3*	−7.0	−5.0	−95.0	−163
*RELA(p65)*	−8.0	−26	−96	−130
*EGFR*	−13	−38	−40.5	−76
*WNT5A*	−1.8	−4.0	1	−94
*REL*	−1.1	−1.8	−1.1	−80

^a^ mRNA ratios BA + STAT3‐inh versus BA.

Overall, STAT3 knockdown induced a pronounced suppression of BA‐induced transcriptional activation of all the analysed genes (Table 1).

### 3.5 Pharmacologic inhibition of STAT3 inhibits BA‐induced transcriptional activation of antiapoptotic and cancer‐related inflammatory genes

**FIGURE 5:**
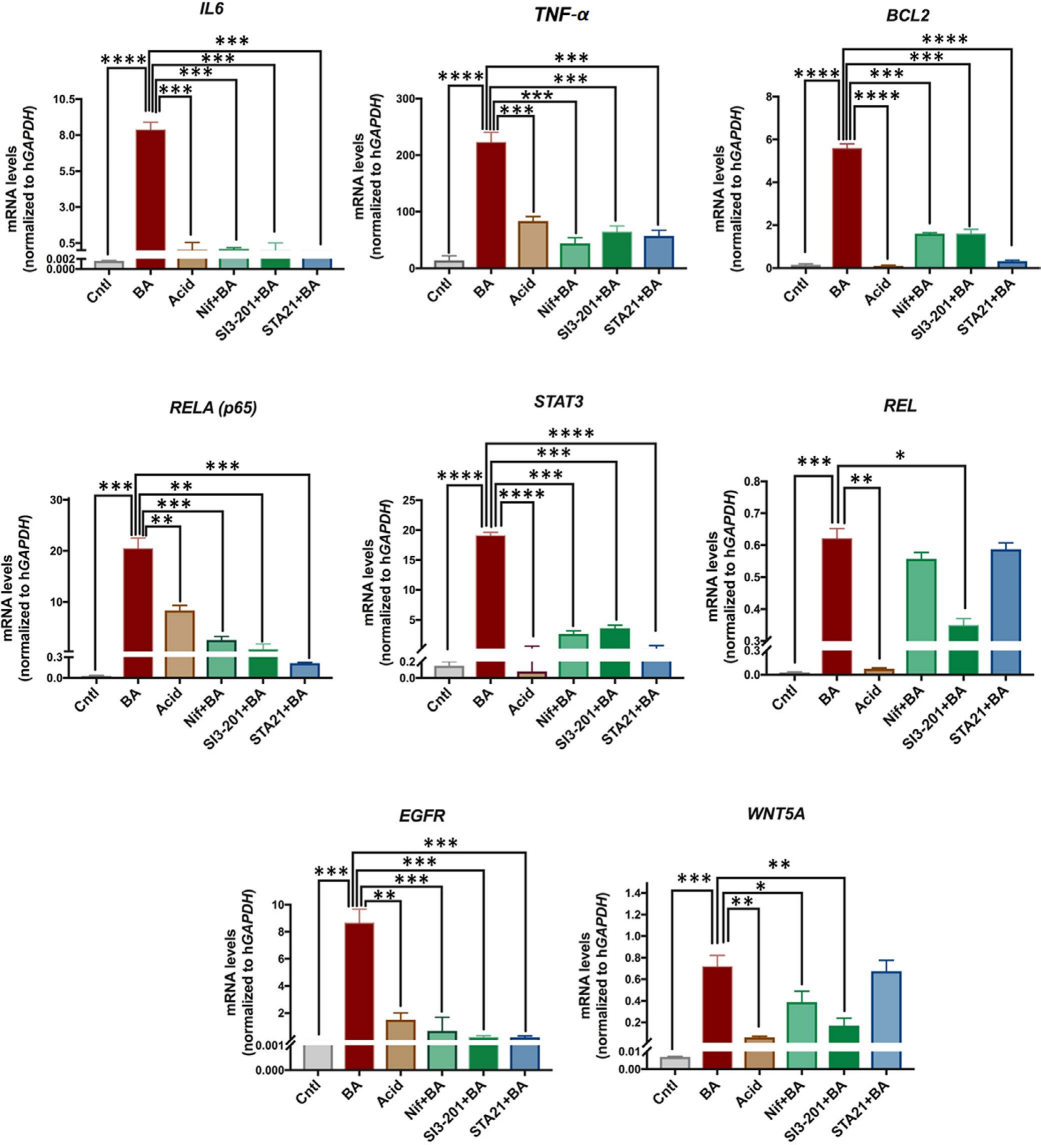
Pharmacologic inhibition of STAT3 inhibits the acidic bile‐induced cancer‐related and inflammatory mRNA phenotype. Columns represent mRNA levels of each analysed gene in treated groups by acidic bile with or without Nifuroxazide, SI3‐201 (STAT3 inhibitor VI) or STA‐21 and controls, by qPCR. (from left to right) *Cntl*: media at pH 7.0 (including vehicle); *BA*: acidic bile (pH 4.0); *Acid*: media at pH 4.0; *Nif*: BA plus Nifuroxazide; *SI3*‐*201*: BA plus STAT3 inhibitor VI (S3I‐201); *STA*‐*21*: BA plus STA‐21. (Normalized mRNAs to *hGAPDH* reference control; mean ± SD of three independent experiments)

qPCR analyses showed that application of Nifuroxazide prevented the BA‐induced mRNA profile of antiapoptotic gene *BCL2*,^32^, ^33^ cancer and inflammatory‐related genes, *IL6*, *TNF*‐*α*, *RELA(p65)*, oncogenic *STAT3* and *EGFR*, as well as of cell proliferation or tumour‐promoting factor *WNT5A*,^8^, ^11^, ^19^, ^26^, ^27^, ^33^, ^34^, ^35^, ^36^, ^37^, ^38^, ^39^, ^40^ as shown in Figure 5 and Table 1 (Supplementary Material; Table S5). Specifically, targeting JAK2/STAT3 phosphorylation, by Nifuroxazide, induced significantly lower mRNA levels of these genes, compared to BA alone.

Application of SI3‐201 similarly prevented the BA‐induced mRNA profile of *TNF*‐*α*, *IL6*, *BCL2*, *RELA(p65)*, *STAT3*, *EGFR* and *WNT5A*, as well as of cell proliferation or tumour‐promoting factors, *REL*,^38^ as shown in Figure 5 and Table 1 (Supplementary Material; Table S5). Specifically, targeting STAT3 dimerization, by SI3‐201, induced significantly lower mRNA levels of these genes, compared to BA.

Finally, as presented in Figure 5 and Table 1, STA‐21 similarly to other two inhibitors prevented the BA‐induced transcriptional changes of *IL6*, *TNF*‐*α*, *BCL2*, *RELA(p65)*, *STAT3* and *EGFR* (Supplementary Material; Table S5). Targeting STAT3 dimerization and its DNA binding, by STA‐21, induced a pronounced reduction of transcriptional levels of *IL6*, *RELA(p65)*, *STAT3* and *EGFR*, compared to BA.

### 3.6 Silencing or pharmacologic inhibition of STAT3 affects cell viability of BA‐treated HCs

**FIGURE 6:**
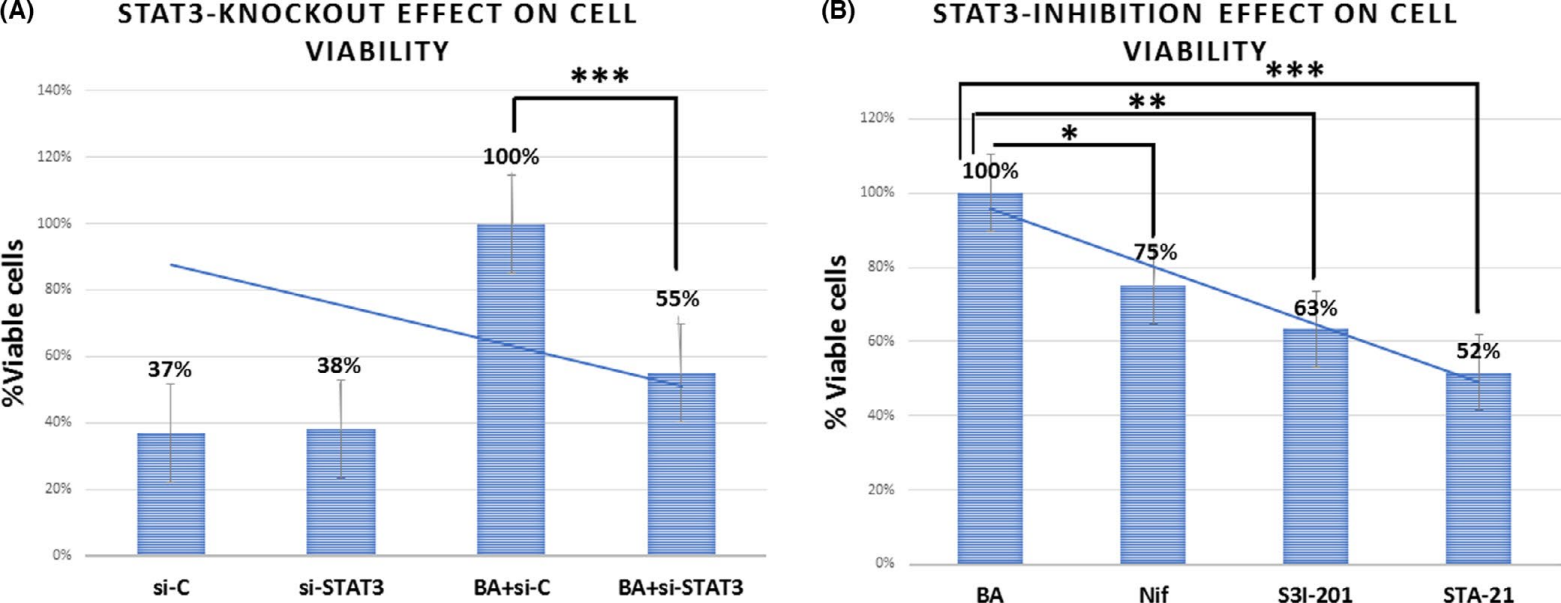
*STAT3* knockout or its pharmacologic inhibition effects on cell viability of BA‐treated HCs (A). Graph depicts differences in cell survival (% viable cells) in BA‐treated HCs with versus without *STAT3* knockout. *si*‐*C*: Control siRNA; *si*‐*STAT3*: STAT3 siRNA; *BA*+*si*‐*C*: BA plus Control siRNA; *BA*+*si*‐*STAT3*: BA plus STAT3 siRNA. (B) Graph depicts the survival rates (% of viable cells) in BA‐treated HCs with versus without STAT3 pharmacologic inhibition (from left to right) *BA*: acidic bile (pH 4.0); *Nif*: BA plus Nifuroxazide; *SI3*‐*201*: BA plus STAT3 inhibitor VI (S3I‐201); *STA*‐*21*: BA plus STA‐21. (*p < 0.05; **p < 0.005, by *t* test; multiple comparisons by Holm‐Sidak; GraphPad Prism 7 software; Data are derived from three independent experiments)

We performed a cell viability assay to explore the effect of *STAT3* knockdown or its pharmacologic inhibition on BA‐induced cell survival rates. Our analysis revealed that silencing *STAT3* (siRNA STAT3; Figure 6A) or blocking STAT3 dimerization and DNA binding, by SI3‐201 or STA‐21, or targeting STAT3 phosphorylation, by Nifuroxazide (Figure 6B) significantly reduced the BA‐induced survival rates of treated HCs (*p* < 0.05; by *t* test; multiple comparisons by Holm‐Sidak). Blocking STAT3 by STA‐21 presented similar to *STAT3* knocking down changes in cell survival of BA‐treated HCs (Figure 6).

## 4 DISCUSSION

Chronic exposure to BA has recently been shown to cause malignant transformation of hypopharyngeal epithelial cells which preceded by significant transcriptional activation of cancer and inflammatory‐related genes, previously assigned as ‘BA‐induced mRNA oncogenic phenotype’, including oncogenic STAT3.^2^, ^3^, ^4^, ^5^ Interestingly, the oncogenic molecular phenotype induced by BA *in vitro* and *in vivo* was similarly identified in clinical specimens from bile‐related HSCC, demonstrating an aberrant overexpression of STAT3 compared to controls.^39^ The role of STAT3 as a crucial transcription factor in HNSCC has been discussed extensively by others.^9^, ^10^, ^20^, ^21^, ^22^, ^27^ Although other STAT factors, such as STAT1 and STAT5, have also been associated with cancers of the upper aerodigestive tract,^42^, ^43^ we focused our current exploration on STAT3, based on our previous findings.^2^, ^3^, ^4^, ^7^, ^12^, ^14^, ^15^, ^16^, ^17^, ^18^ Investigating whether STAT3 contributes substantially to BA‐induced molecular oncogenic profile may elucidate a better understanding to the mechanism of bile reflux‐related hypopharyngeal carcinogenesis and demonstrate useful key molecules for its early detection or targeted treatment. Also, using pharmacologic inhibitors that can block different steps of STAT3 activation process may provide us with insightful information about the upstream signalling of its activation under the BA effect and so make it an attractive tool for future clinical preventive and therapeutic approaches.

Our novel findings conclude that silencing of *STAT3* expression had a dramatic effect on BA‐induced oncogenic molecular phenotype. Specifically, *STAT3* knockdown induced a strong suppression of total p‐STAT3 and Bcl‐2 protein levels, and significantly reduced *STAT3* transcriptional activity and the transcriptional levels of anti‐apoptotic *BCL2*,^32^, ^33^ cancer and inflammatory‐related factors *IL6*, *TNF*‐*α*, *RELA(p65)*, and *STAT3*,^8^, ^11^, ^19^, ^26^, ^27^, ^33^, ^34^, ^35^, ^36^, ^37^, ^38^ or cell proliferation and tumour promotion factors *REL*, *EGFR*, *WNT5A* and *REL*,^36^, ^38^, ^39^, ^40^ previously linked to BA‐induced oncogenic effect.^4^, ^41^ Silencing *STAT3* also reduced BA‐induced cell survival rates. These findings strongly support the hypothesis that STAT3 plays an important role in bile reflux‐related oncogenic molecular events in HCs.^8^, ^9^, ^11^, ^26^, ^27^, ^32^, ^33^, ^34^, ^35^, ^36^, ^37^, ^38^, ^39^, ^40^, ^41^

Our findings from the pharmacologic inhibition of STAT3, using Nifuroxazide,^28^ S3I‐201^22^ or STA‐21,^29^ demonstrated a very similar effect to its gene silencing, supporting their effective application on reducing the BA‐induced oncogenic profile. Using three different inhibitors that each one can block a different step of STAT3 upstream signalling, we gained information that improve our knowledge regarding the mechanism of bile reflux‐related carcinogenesis in hypopharynx. In theory, Nifuroxazide inhibits the JAK/STAT3 phosphorylation, while SI3‐201 and STA‐21 inhibit STAT3 dimerization and its subsequent translocation to the nucleus. As we know, STA‐21 can block STAT3 dimerization, DNA binding and STAT3 dependent transcription, when STAT3 is constitutively active. Herein, we show that all three inhibitors were similarly effective to suppress STAT3 activation, with STA‐21 presenting the most pronounced reduction of BA‐related mRNA phenotype and cell survival. This view may support a constitutive activation of STAT3 in HCs, caused by BA, which does not seem to be exclusively depended on JAK/STAT3 upstream signal,^25^ but also to alternative signalling, such as EGFR.^22^ EGFR cross‐talk with STAT3 has previously been identified to affect activation of STAT3 via tyrosine kinase,^44^ while Bhat et al., recent findings supported EGFR and STAT3 interactions in oesophageal precancerous and cancerous cells under the acidic bile effect.^45^, ^46^ Further investigation is required to elucidate possible similar EGFR‐STAT3 interactions in acidic bile‐related hypopharyngeal squamous cell cancer.^41^

Notably, both SI3‐201 and STA‐21 appeared to produce a relatively more pronounce suppressive effect on BA‐induced mRNA phenotype than Nifuroxazide (Table 1). A possible explanation to this view could be that BA‐induced constitutive activation of STAT3 through JAK/STAT3 pathway may lead to interactions of STAT3 with other STAT factors, like STAT1,^42^, ^43^ contributing to this process. Future exploration of STAT1 in combination with STAT3 inhibitors may clarify the role of STAT in BA‐induced oncogenic effect in hypopharynx.

Besides the above variations, our data demonstrated that similarly to *STAT3* silencing, all three pharmacologic drugs suppressed the transcriptional activation of *IL6*, *TNF*, *STAT3*, *BCL2*, *RELA*(p65) and *EGFR* (Table 1). This observation strongly supports that the constitutive activation of STAT3 can maintain the continuous production of inflammatory and cancer‐related molecules that are central to the carcinogenic process.^4^, ^41^, ^47^ Therefore, we confirm that BA‐induced molecular oncogenic events can be effectively prevented by the application of either Nifuroxazide,^28^ S3I‐201,^22^ or STA‐21.^29^

IL6 plays a central role in inflammatory and cancer promotion of HNSCC and is considered as a promising therapeutic target.^22^, ^25^, ^27^, ^35^ Our current findings showed that BA effect can induce an abundant overexpression of *IL6* in exposed HCs, as previously shown in premalignant or malignant murine hypopharyngeal mucosa and in bile‐related HSCC.^4^, ^41^ *IL6* overexpression can promote a persistent activation of STAT3, by the IL6/STAT3 pathway.^25^, ^35^ Our data show that silencing or pharmacologic inhibition of STAT3 can effectively reduce *IL6* transcriptional levels and thus limit potential positive feedback.

Although we show that targeting STAT3, either by its knockdown or its pharmacologic inhibition, had a minimal effect on total p‐NF‐κB (p65 S536) levels, our findings strongly support a role of STAT3 in promoting the transcriptional activation of NF‐κB. NF‐κB inhibition, using BAY 11‐7082, had previously supported the role of NF‐κB in BA‐induced activation of STAT3, among other factors.^12^, ^13^, ^14^, ^15^, ^16^, ^17^, ^18^ In view of all the above, we propose a possible molecular cross‐talk between the NF‐κB and STAT3 transcription factors signatures in bile reflux‐related inflammation and tumorigenesis in hypopharynx, as has been proposed by others in HNSCC^8^, ^10^, ^11^ that requires further exploration.

Given the central role of STAT3 pathway in HNSCC, despite its complexity, our data provide supportive evidence of STAT3 inhibition as a preventive and therapeutic approach in hypopharyngeal cancer. STAT3 is indeed a demanding target not only because of the complexity of its activating signal but also because of its resistance to standard treatment in HNSCC.^21^, ^22^ However, recent preclinical studies have shown the antiproliferative effect of STAT3 inhibition,^28^, ^48^, ^49^, ^50^, ^51^, ^52^ and the latest clinical trials have presented very promising anti‐oncogenic phenotype under STAT3‐target therapy.^22^ SI3‐201 has been extensively tested as an antitumour target in HNSCC, since it is promising to abrogate resistance to anti‐EGFR therapies.^46^, ^48^, ^49^, ^50^ Nifuroxazide has also been proved to be a safe drug with antitumour and anti‐inflammatory effects.^51^, ^52^ Finally, STA‐21 has been clinically tested and found to improve chronic inflammatory disorders.^53^ Our data from the use of the three pharmacological inhibitors of STAT3 strongly support their use as promising drugs for the prevention or treatment of inflammatory or neoplastic diseases associated with laryngopharyngeal reflux. It was very recently presented an association between bile acid‐receptors, such as nuclear farnesoid X receptors (FXRs),^54^, ^55^ and JAK/STAT3 in colon,^56^ which inspires a further exploration of FXRs possible expression in the hypopharynx and their potential interaction with JAK/STAT3 or other oncogenic pathways in bile reflux‐related HSCC. Other STAT factors, such as STAT1 and STAT5, have also been associated with epithelial cancers and their interaction with STAT3.^42^, ^43^ Future experiments may clarify the role of STAT1 and STAT5 in acidic bile‐induced hypopharyngeal carcinogenesis and how STAT3 silencing, or inhibition may affect their expression.

## 5 CONCLUSION

Our novel data strongly support that STAT3 contributes substantially to bile reflux‐related molecular oncogenic events in HCs. This view was documented by silencing *STAT3* gene which induced a strong suppression of total p‐STAT3 and Bcl‐2 protein levels, and a dramatic reduction of transcriptional levels of cancer‐related cytokines, genes and cell survival. Using three inhibitors, each one targeting a different step of STAT3 upstream signalling, such as Nifuroxazide, S3I‐201 or STA‐21, we extracted novel findings supporting the constitutive activation of STAT3 under the acidic bile effect. This constitutive activation does not appear to be exclusively depended on JAK/STAT3. We also show that all three STAT3 inhibitors, with STA‐21 having the most profound effect, can effectively suppress the continuous production of cancer‐related molecules, *IL6*, *TNF*, *RELA(p65)*, *EGFR*, *BCL2* and *STAT3*, previously associated with hypopharyngeal cancer, caused by acidic bile. These findings document the important role of STAT3 in hypopharyngeal carcinogenesis associated with bile reflux, and also encourage the single or combined application of Nifuroxazide, S3I‐201 or STA‐21, in clinical studies for preventive or therapeutic approaches.

## CONFLICT OF INTEREST

The authors whose names are listed in this article certify that they have no affiliations with or involvement in any organization or entity with any financial or non‐financial interest in the subject matter or materials discussed in this manuscript.

## AUTHOR CONTRIBUTIONS

**Dimitra P Vageli:** Conceptualization (lead); Data curation (lead); Formal analysis (lead); Investigation (lead); Methodology (equal); Project administration (equal); Resources (equal); Software (lead); Supervision (lead); Validation (equal); Visualization (lead); Writing‐original draft (lead); Writing‐review & editing (equal). **Panagiotis G. Doukas:** Data curation (equal); Formal analysis (equal); Investigation (equal); Methodology (equal); Software (equal); Validation (equal); Visualization (equal); Writing‐original draft (equal); Writing‐review & editing (equal). **Athanasios Siametis:** Formal analysis (equal); Methodology (equal); Software (equal); Visualization (equal); Writing‐review & editing (equal). **Benjamin L. Judson:** Data curation (equal); Funding acquisition (lead); Project administration (equal); Resources (equal); Validation (equal); Writing‐review & editing (equal).

## Supporting information

Supinfo S1

## Data Availability

Data are contained within the article or Supplementary Material.
