# Supplementary material for: Targeting STAT3 prevents bile reflux‐induced oncogenic molecular events linked to hypopharyngeal carcinogenesis
Source: J Cell Mol Med. 2021 Dec 1;26(1):75–87. doi: 10.1111/jcmm.17011 (PMC8742186; doi:10.1111/jcmm.17011)
Supplement: Supplementary file 1 — Supinfo S1 [file JCMM-26-75-s001.docx]

**Title: Silencing or pharmacologic inhibition of STAT3 prevents early molecular oncogenic events of bile reflux-related hypopharyngeal carcinogenesis.**

Dimitra Vageli^1^, Panagiotis G. Doukas^1^, Athanasios Siametis^1^, Benjamin Judson^1^

^1^The Yale Larynx Laboratory, Department of Surgery (Otolaryngology), Yale School of Medicine, New Haven, CT, USA.

**Supplementary methods**

*Luciferase assay*

Luciferase assay was performed in Hypopharyngeal primary cells (HC) exposed to acidic bile with pharmacologic inhibitors of STAT3, or STAT3 knockout, compared to HC exposed to acidic bile alone and controls. A STAT3 dual-luciferase reporter assay was used (Cignal reporter assay by Qiagen, Germantown, MD, USA), including (i) a firefly luciferase reporter for STAT3 and a constitutively expressing Renilla luciferase construct (Creport-STAT3), and (ii) a cignal negative control with a non-inducible reporter construct and a constitutively expressing Renilla luciferase construct (Creport-NC). We performed a reverse transfection using Lipofectamine® 2000 (Invitrogen™, Waltham, MA, USA) according to manufacturer’s procedure.

*Luciferase assay in HC exposed to acidic bile with pharmacologic inhibitors of STAT3:* Briefly, HC were seeded in medium with serum without antibiotics (Opti-MEM® serum-reduced growth medium, Gibco™ by Thermo Fisher Scientific, Franklin, MA, USA). Sixteen hours after transfection the medium was changed to complete growth medium with antibiotics. The treatment was performed 24 hours after transfection. The cells were treated with bile at pH 4.0 with or without inhibitors and controls, for 20-25 min and then media were replaced with serum free medium. After 4-6 hours incubation luciferase assay was developed by using a firefly Luciferase Assay system (Promega Corporation, Madison, WI, USA), following the manufacturer’s protocol and luminescence was measured using a luminometer (Synergy1, BioTek Instruments Inc., Winooski, VT, USA) and Gen5 software. We performed triplicate assays for each treatment condition.

*Luciferase assay in acidic bile-treated HC with knockout of STAT3:* STAT3 siRNA (si-STAT3) (sc-29493; Santa Cruz Biotechnology Inc., Dallas, TX, USA) (5 nM in serum free) was pre-mixed with STAT3 luciferase reporter (Creport STAT3; Cignal reporter assay, Qiagen, Germantown, MD, USA), and Lipofectamine® 2000 (Invitrogen™, Waltham, MA, USA), according to manufacturer’s procedure. A Control siRNA (si-Control) (sc-37007; Santa Cruz Biotechnology Inc., Dallas, TX, USA) was also used as a reference control. Sixteen hours after transfection media were changed with complete growth medium and four hours later were treated with acidic bile for 20-25 min. Then the media were removed and the cells were incubated in serum free medium. After 6 hours of incubation a luciferase assay was developed by using a firefly Luciferase Assay system (Promega Corporation, Madison, WI, USA), following the manufacturer’s protocol. Luminescence was measured using a luminometer (Synergy1, BioTek Instruments Inc., Winooski, VT, USA) and Gen5 software. Assays were carried out according to the manufacturer ’s instructions and performed in triplicate. All experiments were independently repeated two times.

*Immunofluorescence Cell Staining*

HC were grown on multiwall chamber slides; Lab-Tek^®^ and treated with acidic bile at pH 4.0, with or without pharmacologic inhibitor of STAT3 and controls at pH 4.0 (acid control) or at neutral pH 7.0 (control including vehicle) (Table S3). Cells were fixed immediately after the final exposure to experimental or control media in 4% paraformaldehyde for 7 min, followed by 3 washes with PBS, permeabilization of cell membranes using 0.2% Triton X100-PBS (AmericanBio, Natick, MA, *USA*) for 3 min, and blocking with 2% bovine serum albumin (BSA)-PBS (Sigma-Aldrich, by MilliporeSigma, St. Louis, MO, USA) for 1 h. All groups were then incubated with 1:65 of primary anti-p-STAT3 (Tyr705) (rabbit mAb, D3A7 XP^®^, Cell Signaling Technology, Inc., MA, USA) or anti-phospho-NF-*κ*B (rabbit polyclonal anti-phospho-p65 Ser536, AbD Serotec, Bio-Rad, Hercules, CA, USA) overnight at 4 °C. Cells were washed by 1% Tween-PBS and incubated with 1:500 dilutions of secondary anti-rabbit or or anti-mouse DyLight^®^488 (green; Vector Labs, Burlingame, CA, USA), for 1 h, at room temperature. Followingly, cells were mounted by Prolong Gold Mountant with diamidino-phenylindole (ProLong^®^ Diamond Antifade Mountant with DAPI; Life Technologies, Thermo Scientific, Franklin, MA, USA) for nuclear staining (blue color). Zeiss Confocal microscope and Zen imaging software were used to examine stained slides and captured images, respectively (Zen imaging software, Carl Zeiss, microscopy GmbH, Jena, Germany) [6,7]. Expression levels of p-STAT3 and p-NF-kB were identified by fluorescence intensity (mean±SD bin count) from at least two intendent images (>10 cells) (Zen imaging software).

*Protein expression analyses*

At the end of treatment, cultured HC were harvested, and washed once with PBS. Total protein was isolated using M-PER reagent (mammalian protein extraction reagent; Thermo Scientific), while and cytoplasmic and nuclear fractions were isolated by NE-PER nuclear and cytoplasmic extraction, including protease inhibitors (Thermo Scientific, Pierce, NY). Total, cytoplasmic or nuclear protein concentrations were determined using BCA-200 Protein Assay kit (Thermo Fisher Scientific, Waltham, MA, USA).

*Western blot analysis*: Twenty to 30 µg of total or nuclear and cytoplasmic protein extracts from experimental and control-treated HC were heated at 70 °C for 10 min in sodium dodecyl sulfate-polyacrylamide gel electrophoresis Laemmli sample buffer (Bio-Rad, Hercules, CA, USA), and separated using 4-20% Mini-PROTEAN TGX Tris/Glycine precast gels, at 150V for 1 h. Precision plus pre-stained protein standards (Dual Color or Kaleidoscope, Bio-Rad, Hercules, CA, USA) were used as molecular-weight size markers. Proteins were transferred onto a 0.45 mm nitrocellulose membrane, using a Trans-Blot Turbo transfer system (Bio-Rad, Hercules, CA, USA). After blocking in 5% BSA, for 1 h, membranes were incubated with primary antibodies, 1:1000 of STAT3 (clone F-2), 1:1000 bcl2 (Clone N-19), 1:500 of p-STAT3 (Tyr 705) (clone B-7), and 1:500 p-NF-kB p65 Antibody (27.Ser 536) (Santa Cruz Biotechnology Inc., Dallas, TX, USA), which were diluted in 5% BSA, overnight at 4 °C. Membranes were incubated for 1:30 h with goat anti-mouse horseradish peroxidase-conjugated secondary antibodies (EMD Millipore, Burlington, MA, USA) at 1:3000, and chemiluminescence was determined using an enhanced chemiluminescence detection system (Clarity Western ECL Substrate, Bio-Rad). Membranes were also stripped using Restore^TM^ Western Blot Stripping buffer (Pierce Biotechnology, Rockford, IL, USA) and reported with β-actin (C4; Santa Cruz Biotechnology Inc., Dallas, TX, USA) for cytoplasmic extracts and Histone 1 (AE-4; Santa Cruz Biotechnology Inc., Dallas, TX, USA) for nuclear extracts normalization. Protein levels were quantified by the Gel imaging system (Bio-Rad, Hercules, CA, USA) in total protein extracts or in each nuclear or cytoplasmic cellular compartment (Image Lab 5.2 analysis software, Bio-Rad, Hercules, CA, USA).

*Enzyme-Linked Immunosorbent Assay for Total p-STAT3, p-NF-κB and bcl-2 Quantification:* We performed a direct enzyme-linked immunosorbent assay (ELISA) to quantify cytoplasmic bcl2, and nuclear p-NF-kB, and p-STAT3 expression levels. Nunc MaxiSorp™ 96-well plates (Invitrogen™, Waltham, MA, USA) were coated with 100 μL of cytoplasmic and nuclear protein extracts from human HC, at a concentration of 10 μg/mL, in 1X coating buffer [protein added to coating buffer and mixed for 15 min; 1X coating buffer diluted from 5X stock (BUF030A; Bio-Rad, Hercules, CA, USA) in dH_2_O and mixed for 15 min]. The plates were covered and incubated at 4 °C overnight. The next day, the plates were washed 3 times in wash buffer (PBST; 0.05% v/v Tween-20 in PBS), incubated in 150 μL/well of blocking solution (1% w/v BSA in PBS) for 60 min at 37 °C, and then washed 4 times in wash buffer and incubated for 1 h at 37 °C, in 100 μL/well of primary p-STAT3 (clone B-7), p-NF-*κ*B (p65 Antibody 27.Ser 536), bcl2 (Clone N-19), and β-actin (Clone C4; which was used as a reference control for total protein normalization) mouse monoclonal antibodies HRP (Santa Cruz Biotechnology Inc., Dallas, TX, USA). (An amount of 0.3 μg of each antibody was used per 1-2 μg of protein; antibodies were diluted in 1%BSA/PBS). Finally, the plates were washed 3 times in wash buffer and incubated in 100 μL/well of TMB Core+ substrate solution (3,3′, 5, 5′-tetramethylbenzidine plus hydrogen peroxide) (BUF062C; Bio-Rad, Hercules, CA, USA) for 30 min at room temperature in the dark. The absorbance values were immediately measured at 600 nm using a microplate reader (Sunergy1, BIOTEK; Gen5^TM^ software, BioTek Instruments Inc., Winooski, VT, USA). Protein standards for β-actin were used by 1:10 serial dilutions of a highly concentrated protein sample that was positive for p-STAT3. Assays were carried out according to the manufacturer’s instructions and performed in triplicate. All experiments were repeated three times, independently.

*Quantitative Real-Time Polymerase Chain Reaction*

Quantitative real-time polymerase chain reaction (qPCR) analysis was performed for *EGFR, TNF-a, IL6, STAT3, RELA(p65), cREL, BCL2 and WNT5A*. Total RNA was isolated (RNeasy mini kit; Qiagen Inc., Valencia, CA, USA) from experimental and control treated HC. RNA quality and concentration were evaluated by absorption ratios 260/280 nm (>2.0) and 260 nm, respectively (NanoDrop^TM^ 1000 spectrophotometer; Thermo Fisher Scientific, Waltham, MA, USA). Subsequently, reverse transcription (iScript cDNA synthesis kit; Bio-Rad, Hercules, CA, USA) from total RNA and real-time qPCR analysis (Bio-Rad real-time thermal cycler CFX96^TM^; Bio-Rad, Hercules, CA, USA) were performed, using specific primers for target genes and reference housekeeping gene, human glyceraldehyde 3-phosphate dehydrogenase (*h*GAPDH) (QuantiTect Primers Assays; Qiagen, Germantown, MD, USA) (Supplementary materials; **Table S3**) and iQ^TM^ SYBR Green Supermix (Bio-Rad, Hercules, CA, USA). Our assay was performed on 96-well plates, in triplicates, and data were analyzed by CFX96^TM^ software [6,7,11,16]. Relative mRNA expression levels were estimated for each target gene compared to the reference control gene (ΔΔ*C*t).

**Supplementary Table S1**: Experimental and control groups of pharmacologic inhibition of STAT3.

|  | **Cntl** | **Acid** | **BA** | **Nif** | **SI3-201** | **STA-21** |
| --- | --- | --- | --- | --- | --- | --- |
| **Media pH 7.0* | **+** |  |  |  |  |  |
| *Media pH 4.0* |  | **+** | **+** | **+** | **+** | **+** |
| *Bile* | **-** | **-** | **+** | **+** | **+** | **+** |
| *Nifuroxazide* | **-** | **-** | **-** | **+** | **-** | **-** |
| *STAT3 inhibitor VI (S3I-201)* | **-** | **-** | **-** | **-** | **+** | **-** |
| *STA21* | **-** | **-** | **-** | **-** | **-** | **+** |

*containing vehicle (DMSO)

**Supplementary Table S2**: Experimental and control groups of STAT3 knockdown.

|  | **si-C** | **si-STAT3** | **BA+si-C** | **BA+si-STAT3** | **Acid+si-C** | | **Acid+si-STAT3** |
| --- | --- | --- | --- | --- | --- | --- | --- |
| *Media pH 7.0* | **+** | **+** |  |  |  |  | |
| *Media pH 4.0* |  |  | **+** | **+** | **+** | **+** | |
| *Bile* | **-** | **-** | **+** | **+** | **-** | **-** | |
| *STAT3 siRNA* | **-** | **+** | **-** | **+** | **-** | **+** | |
| *Control siRNA* | **+** | **-** | **+** | **-** | **+** | **-** | |

**Supplementary Table S3**: Human genes analyzed by real-time qPCR, in human hypopharyngeal cancer cells (HC).

| **Gene** | **Detected transcripts** | **Amplicon length (bp)** |
| --- | --- | --- |
| ***h*GAPDH** | NM_001256799, NM_002046 | 95 |
| ***BCL2*** | NM_000633 | 116 |
| ***EGFR*** | NM_005228  NM_201282-4, | 80 |
| ***RELA*** | NM_001145138, NM_001243984-5, NM_021975 | 107 |
| ***WNT5A*** | NM_001256105, NM_003392 | 105 |
| ***TNF-α*** | NM_000594 | 98 |
| ***STAT3*** | NM_003150,  NM_139276 | 95 |
| ***IL6*** | NM_000600 XM_005249745 | 107 |
| ***REL*** | NM_002908 | 117 |

**Supplementary Table S4:** Silencing STAT3-induced transcriptional levels of anti-apoptotic or cancer-related genes in control and acidic bile-treated human HC.

| **Target gene/**  ***hGapdh** (*ΔΔ^CT^*)** | **Cntl** | | **BA** | | **Acid** | |
| --- | --- | --- | --- | --- | --- | --- |
|  | **si-C** | **si-STAT3** | **si-C** | **si-STAT3** | **si-C** | **si-STAT3** |
| *TNF-α* | 1.40E-04 | 1.80E-04 | 2.93E+01 | 1.25E-01 | 9.30E-03 | 8.90E-03 |
| *RELA(p65)* | 5.10E-04 | 5.00E-04 | 4.81E+01 | 3.70E-01 | 4.00E-01 | 3.50E-01 |
| *IL6* | 1.00E-03 | 8.80E-04 | 1.43E+01 | 4.59E-02 | 3.00E-03 | 1.80E-03 |
| *EGFR* | 1.40E-05 | 1.00E-05 | 2.27E-01 | 2.99E-03 | 2.90E-05 | 2.40E-05 |
| *STAT3* | 2.58E-04 | 1.18-04 | 1.27E+00 | 3.77E-04 | 5.00E-04 | 3.32E-04 |
| *BCL2* | 4.32E-02 | 4.16E-02 | 1.71E+02 | 6.00E-01 | 2.40E-02 | 2.00E-02 |
| *REL* | 3.00E-05 | 2.80E-05 | 4.00E-01 | 5.02E-03 | 1.40E-05 | 1.10E-05 |
| *WNT5A* | 6.99E-05 | 9.00E-05 | 5.80E-01 | 6.18E-03 | 9.30E-04 | 4.50E-04 |

* normalization of mRNA levels using *hGAPDH; si-C: Control siRNA; si-STAT3: STAT3 siRNA; BA: Acidic bile (pH 4.0); Cntl: Control-DMSO (pH 7.0); Acid (pH 4.0).*

**Supplementary Table S5.**  Pharmacologic inhibition of STAT3-induced transcriptional levels of anti-apoptotic or cancer-related genes in acidic bile-treated human HC.

| **Target gene/**  ***hGapdh** (*ΔΔ^CT^*)** | **Control** | **Acid** | **BA** | **Nif** | **SI3-201** | **STA-21** |
| --- | --- | --- | --- | --- | --- | --- |
| *TNF-α* | 1.42E+01 | 8.34E+01 | 2.23E+02 | 4.41E+01 | 6.50E+01 | 5.72E+01 |
| *RELA(p65)* | 2.58E-02 | 2.33E-01 | 2.05E+01 | 2.53E-01 | 7.80E-01 | 2.14E-01 |
| *IL6* | 1.59E-03 | 4.09E-02 | 8.39E+00 | 8.18E-02 | 1.40E-02 | 5.40E-03 |
| *EGFR* | 1.40E-03 | 1.50E+00 | 8.67E+00 | 6.79E-01 | 2.30E-01 | 2.14E-01 |
| *STAT3* | 1.10E-01 | 1.80E-01 | 1.91E+01 | 2.66E+00 | 3.60E+00 | 2.01E-01 |
| *BCL2* | 1.53E-01 | 9.07E-02 | 5.60E+00 | 1.60E+00 | 1.61E+00 | 3.20E-01 |
| *REL* | 2.59E-02 | 7.46E-02 | 6.22E-01 | 5.57E-01 | 3.50E-01 | 5.87E-01 |
| *WNT5A* | 6.99E-03 | 6.18E-02 | 7.21E-01 | 3.89E-01 | 1.70E-01 | 6.70E-01 |

* normalization of mRNA levels using *hGAPDH; Control: media at pH 7.0 with DMSO;* BA: Acidic bile (pH 4.0); Acid: media at pH 4.0; Nif: BA plus Nifuroxazide; SI3-201: BA plus STAT3 inhibitor VI (SI3-201); STA-21: BA plus STA-21
